# Supplementary figures and images for: The ubiquitin-like modifier FAT10 does not affect IL-12 expression and signaling
Source: PLoS One. 2025 May 6;20(5):e0323005. doi: 10.1371/journal.pone.0323005 (PMC12054880; doi:10.1371/journal.pone.0323005)

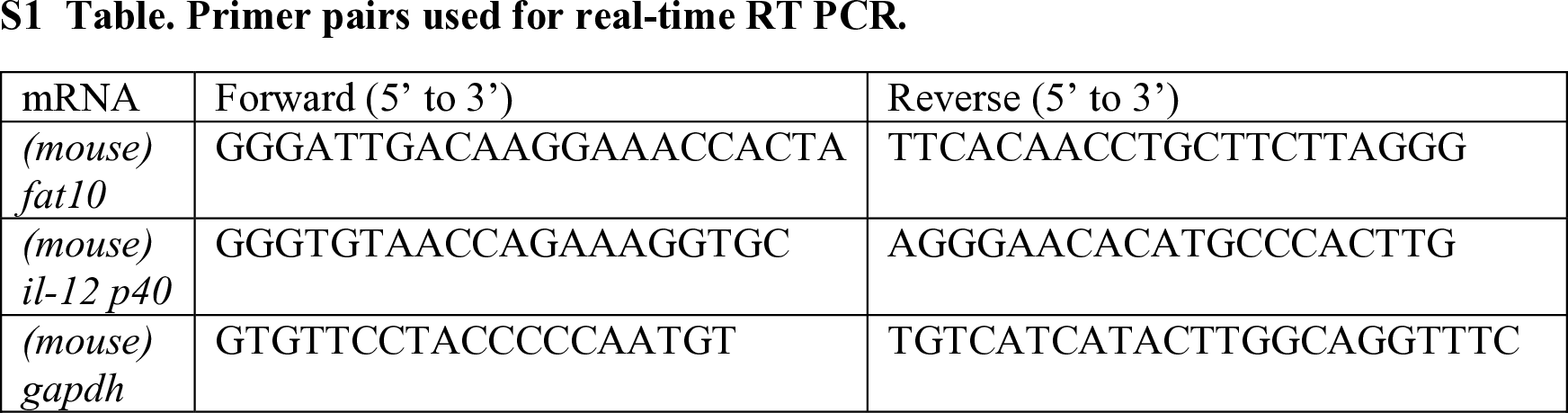

Supplement: S1 Table — (TIF) [file pone.0323005.s001.tif]

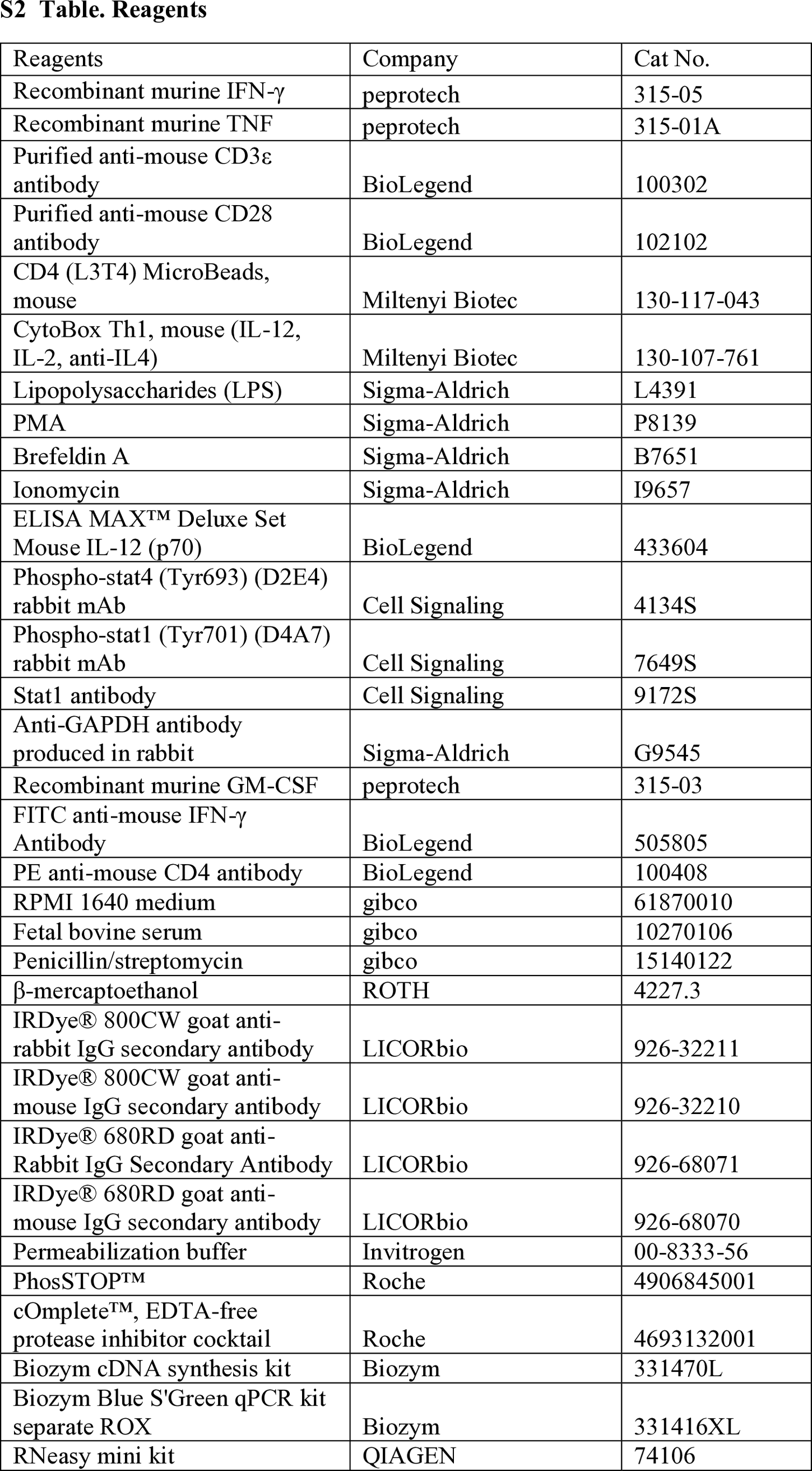

Supplement: S2 Table — (TIF) [file pone.0323005.s002.tif]

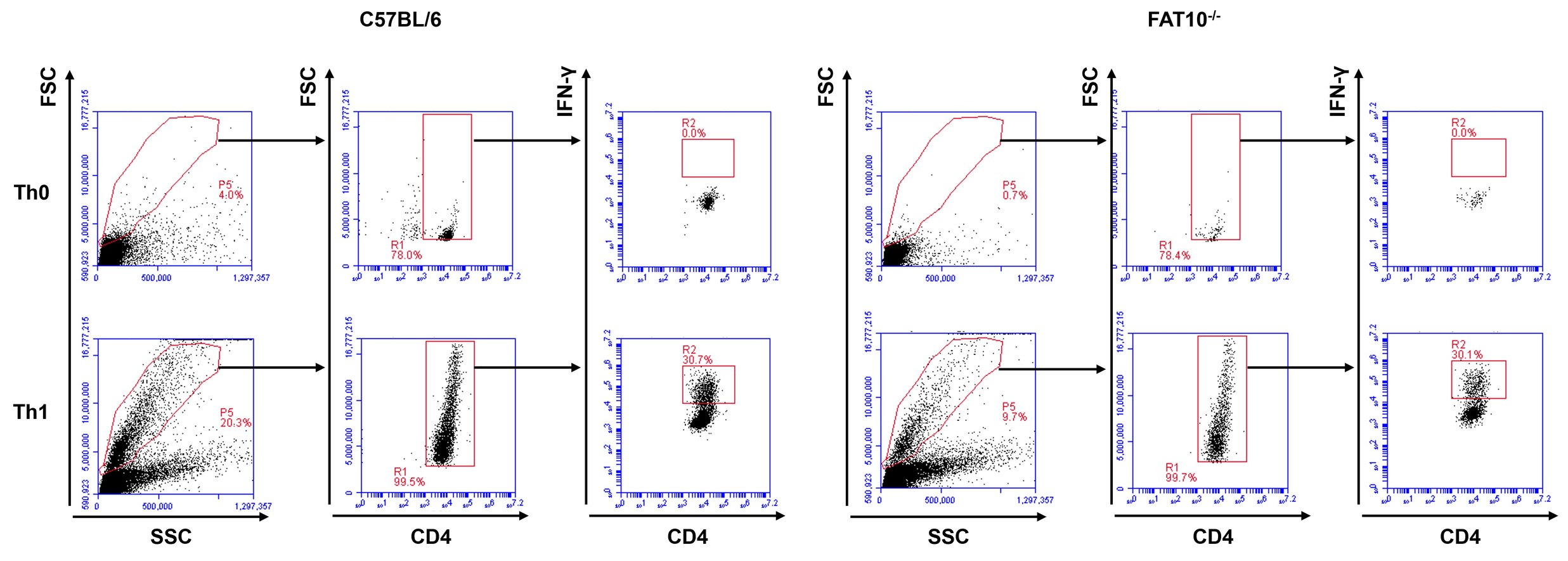

Supplement: S1 Fig — (TIF) [file pone.0323005.s003.tif]

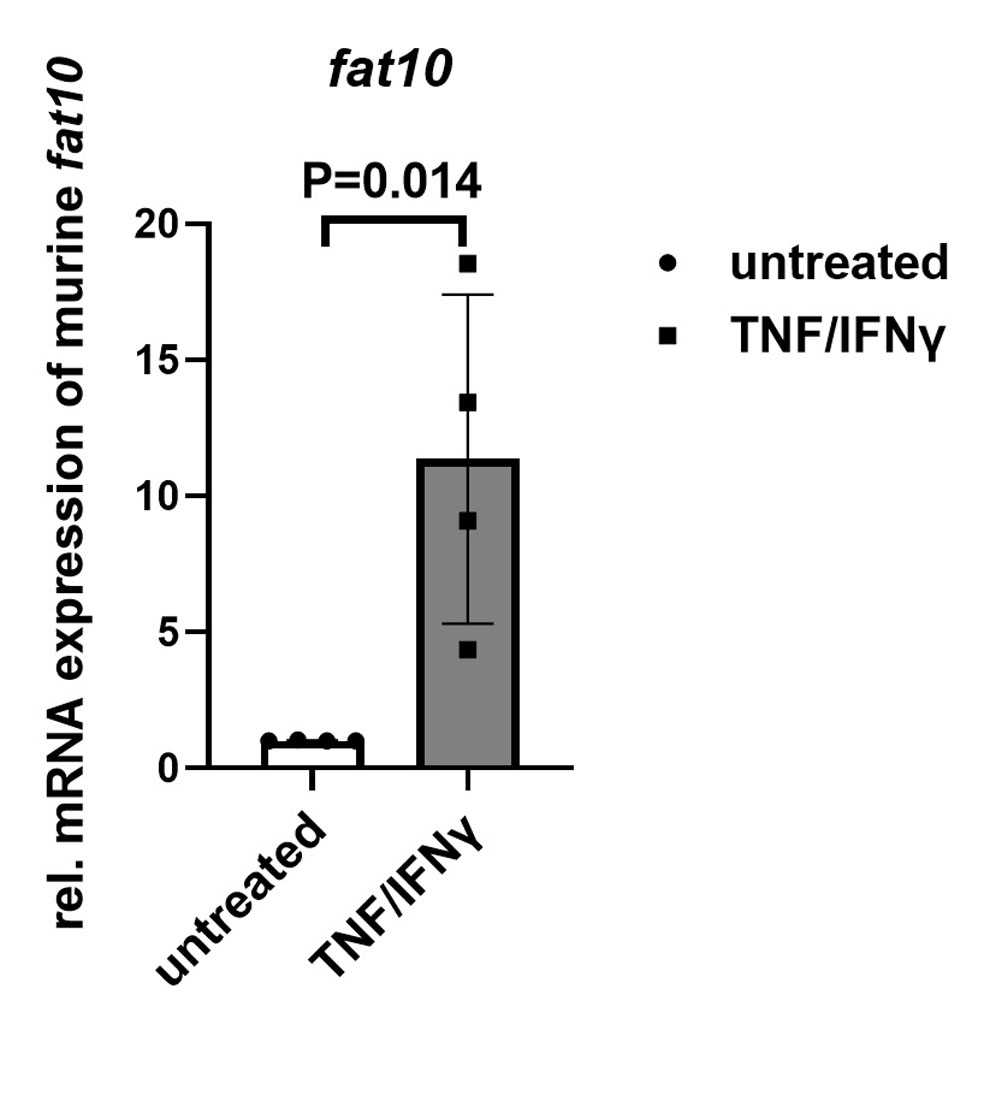

Supplement: S2 Fig — CD4+ T cells were treated with TNF (400 U/ml)/IFN-γ (200 U/ml) (indicated +) for 1 day or were left untreated (indicated -). The mRNA level of FAT10 were determined by real-time RT-PCR. The y-axis depicts relative mRNA levels normalized to GAPDH. Data are depicted as mean ± SDs derived from 4 different experiments (n = 4). (TIF) [file pone.0323005.s004.tif]

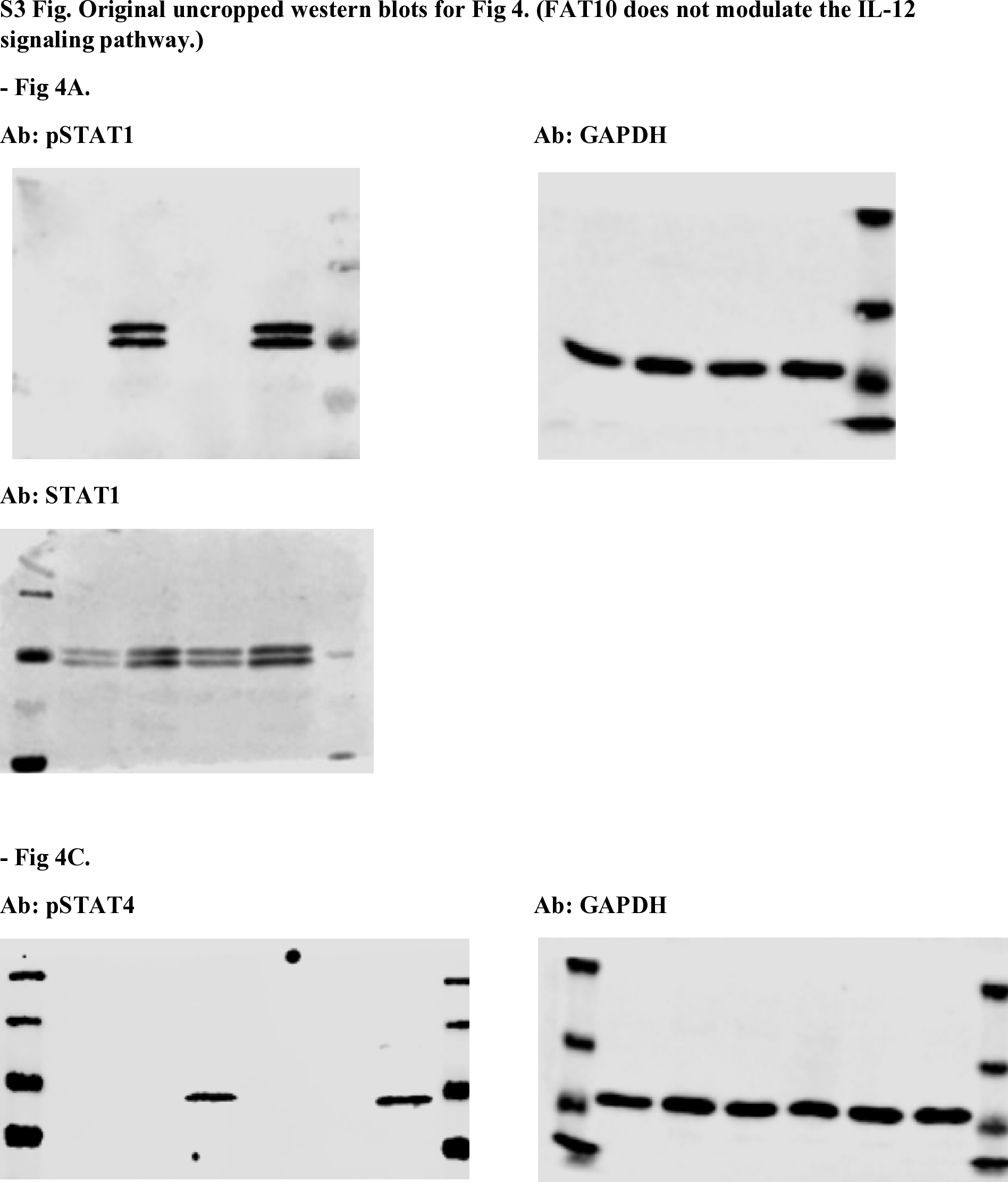

Supplement: S3 Fig — (TIF) [file pone.0323005.s005.tif]
